# Macrophages promote Fibrinogenesis during kidney injury

**DOI:** 10.3389/fmed.2023.1206362

**Published:** 2023-06-22

**Authors:** Hanna Oh, Ohbin Kwon, Min Jung Kong, Kwon Moo Park, Jea-Hyun Baek

**Affiliations:** ^1^Laboratory of Inflammation Research, Handong Global University, Pohang, Gyeongbuk, South Korea; ^2^School of Life Science, Handong Global University, Pohang, Gyeongbuk, South Korea; ^3^Department of Anatomy, BK21Plus, Cardiovascular Research Institute, School of Medicine, Kyungpook National University, Daegu, South Korea

**Keywords:** kidney fibrosis, chronic kidney disease, macrophages, coagulation factor, fibrinogenesis

## Abstract

Macrophages (Mø) are widely considered fundamental in the development of kidney fibrosis since Mø accumulation commonly aggravates kidney fibrosis, while Mø depletion mitigates it. Although many studies have aimed to elucidate Mø-dependent mechanisms linked to kidney fibrosis and have suggested various mechanisms, the proposed roles have been mostly passive, indirect, and non-unique to Mø. Therefore, the molecular mechanism of how Mø directly promote kidney fibrosis is not fully understood. Recent evidence suggests that Mø produce coagulation factors under diverse pathologic conditions. Notably, coagulation factors mediate fibrinogenesis and contribute to fibrosis. Thus, we hypothesized that kidney Mø express coagulation factors that contribute to the provisional matrix formation during acute kidney injury (AKI). To test our hypothesis, we probed for Mø-derived coagulation factors after kidney injury and uncovered that both infiltrating and kidney-resident Mø produce non-redundant coagulation factors in AKI and chronic kidney disease (CKD). We also identified F13a1, which catalyzes the final step of the coagulation cascade, as the most strongly upregulated coagulation factor in murine and human kidney Mø during AKI and CKD. Our *in vitro* experiments revealed that the upregulation of coagulation factors in Mø occurs in a Ca^2 +^ −dependent manner. Taken together, our study demonstrates that kidney Mø populations express key coagulation factors following local injury, suggesting a novel effector mechanism of Mø contributing to kidney fibrosis.

## Introduction

Kidney fibrosis is an irreversible outcome that is a hallmark of chronic kidney disease (CKD). Mounting evidence has demonstrated that Mø play a key role in kidney fibrosis. In this context, many studies have shown that the accumulation of kidney Mø correlates with the severity of kidney injury and fibrosis, while the depletion of kidney Mø reduces fibrosis (1–9). For this reason, researchers have been searching for Mø-dependent mechanisms promoting kidney fibrosis. Previously, it was shown that (1) Mø promote extracellular matrix formation, (2) produce fibrosis-related matrix metalloproteases, (3) secrete profibrotic cytokines, and (4) directly transdifferentiate to myofibroblasts (10–13). However, most of the proposed roles can also be attributed to other effector cells (e.g., myofibroblast), and the latter role is even controversial (14). Of note, Mø are versatile, heterogeneous immune cells and are broadly subdivided into M1 (cyto-destructive) and M2 (tissue-reparative) Mø, which are widely accepted as anti- and profibrotic cells, respectively (15–20). The effects of each Mø subpopulation on fibrosis have given rise to debates (8). Overall, previous studies have not fully captured the key pathogenic role of Mø in kidney fibrosis.

Recently, researchers have identified Mø as a critical source of coagulation factors under certain pathological conditions. Tumor-associated Mø (TAMs) are found to synthesize coagulation factors (F) 7 and 10 (21, 22), and F13a synthesized by monocytes and Mø to impede antitumor immunity in the tumor microenvironment (23). Another study pinpointed myocardial Mø as a major source of circulating F13a (24). In 2019, Zhang et al. suggested that resident peritoneal Mø produce F5 and other clotting factors that are central to host defense in the peritoneum (25).

Previously, it became evident that the coagulation cascade is directly associated with fibrotic development in major organs (e.g., lung, liver, heart, and kidney) (26). In line with this, numerous studies have shown that fibrinogenesis increases fibrotic development while fibrinolysis prevents fibrosis (27–33). Consequently, we reasoned that kidney Mø are the crucial source of coagulation factors that induce fibrinogenesis, ultimately contributing to renal fibrosis. Therefore, in this study, we tested the hypothesis that kidney-resident Mø express key coagulation factors contributing to the provisional matrix formation after a local injury.

## Methods

### Mice

All animal experiments were approved by the Handong Global University Animal Care and Use Committee (Approval No. HGUIACUC20211214-18). C57BL/6 J (B6) female mice were purchased from Hyochang Science, Inc. (Daegu, South Korea) and maintained in a temperature and humidity-controlled environment on a 12 h dark/light cycle.

### Renal I/R

Female mice (6–9 weeks of age) were anesthetized with ketamine/xylazine (60 and 12 mg, respectively per kg body weight). Ischemia was induced by clamping the renal artery of the right kidney with nontraumatic microaneurysm clamps (Roboz Surgical Instrument, Gaithersburg, MD) for 45 min. During ischemia, body temperature was maintained at 36.8–37.2°C by placing mice on a heating pad. Mice were euthanized on days 0, 1, 6, or 20 following surgery and 40–50 mL of cold phosphate-buffered saline was administered through the left ventricle.

### Renal histology

Kidney tissues were fixed in 4% paraformaldehyde and were embedded in paraffin. Serial 4-μm sections were stained with hematoxylin and eosin (H&E) and periodic acid-Schiff (PAS) to assess the renal injury, and von Kossa staining for calcium deposits. Fibrosis was assessed by Picosirius Red staining and quantifying collagen deposition (red staining) using ImageJ software (National Institute of Health, Bethesda, MD) in 5 randomly selected fields in the section of each group.

### Real-time quantitative PCR

Total RNA was extracted using MiniBEST™ Universal RNA Extraction Kit (Takara Bio Inc., Shiga, Japan). Reverse transcription reaction was performed using Primescript™ 1st strand cDNA synthesis kit (Takara Bio Inc). Quantitative real-time PCR was performed using GoTaq™ qPCR Master Mix (Promega, Madison, WI) on a StepOnePlus™ Real-Time PCR system (Applied Biosystems, Foster City, CA). The amplification conditions were 95°C for 2 min, followed by 40 PCR cycles of 95°C for 15 s and 60°C for 1 min with SYBR green fluorescence detection. Primers are listed in Supplementary Table 1. The gene expression results were normalized to the expression of *Gapdh*, and the *ΔΔCt* method was used for calculating relative expression levels.

### Western blot

Western blot was performed as previously described (34). Kidney protein was extracted using a PRO-PREP™ protein extraction solution (iNtRON Biotechnology, Seongnam, South Korea), and 25 μg total protein was used for western blot. Proteins were separated by 10% SDS-PAGE and transferred to PVDF membranes. The membranes were blocked with 5% skim milk in TBS-T at room temperature for 1 h, then were probed with rabbit anti-mouse/human polyclonal F10/10a Ab or F13A (Invitrogen, Waltham, MA). Mouse monoclonal Direct-Blot HRP anti-GAPDH Ab (BioLegend, San Diego, CA) was used as a loading control. The proteins were visualized by ECL substrate solution and captured using a chemiluminescent imaging system (Azure 280, Azure Biosystems, Dublin, CA), and densitometric analyses were done using ImageJ software (National Institute of Mental Health, Bethesda, MD).

### Serum F13a1 analysis

Fresh blood was collected in a BD Microtainer SST tube (BD Scientific, Franklin Lakes, NJ) and allowed to clot for a minimum of 30 min. Separated serum was frozen at -80’C until analysis. Serum F13a1 level was assayed using Mouse F13a1/F13A chain ELISA kit (ABclonal, Woburn, MA).

### Immunofluorescence

Kidney tissues were frozen in the OCT compound and stored at −80°C before processing. Serial 5-μm cryosections were stained for the presence of macrophages, using FITC-labeled rat anti-mouse CD68 or Alexa Fluor 488-labeled rat anti-mouse CD206 antibodies (BioLegend); and coagulation factors using FITC anti-mouse/human polyclonal F10/10a Ab or F13a (Invitrogen) followed by Alexa Fluor 568-labeled Goat anti-rabbit IgG (Invitrogen). Slides were mounted with a ProLong™ Gold Antifade Mountant with DAPI (Thermo Fisher Scientific, Waltham, MA). Images were taken using immunofluorescence microscopy (Carl Zeiss Axio Imager a2, Oberkochen, Germany) and processed with ImageJ software (National Institute of Mental Health, Bethesda, MD).

### Flow cytometry analysis

Kidney tissues were digested in RPMI medium, 0.1 mg/mL collagenase IV at 37°C for 1 h. Tissues were disaggregated by aspiration through 20G syringes and filtered through a 70-μm cell strainer. Cells were stained with PerCP/Cyanine5.5-labeled rat anti-mouse CD45 (clone: 30-F11), PE/Cyanine7-labeled rat anti-mouse/human CD11b (clone: M1/70), PE/Dazzle 594-labeled rat anti-mouse Ly6G (clone: 1A8), and FITC-labeled rat anti-mouse F4/80 (clone: BM8) antibodies by surface staining. Cells were washed with staining buffer (PBS, 0.5% w/v BSA, 0.01% w/v sodium azide) and fixed with 4% paraformaldehyde in PBS and permeabilized with 0.1% Triton X-100 (Sigma-Aldrich, St. Louis, MO) in PBS before staining with rabbit anti-mouse/human polyclonal F10/10a Ab or F13A (Invitrogen) followed by PE-labeled Donkey anti-rabbit IgG. Flow cytometry analysis was operated using an Attune NXT (Thermo Fisher Scientific) and the data were analyzed using FlowJo software v10.8 (BD Biosciences, Franklin Lakes, NJ). Unless otherwise stated, all antibodies were purchased from BioLegend.

### RNAseq data analysis

Differential gene expression data were obtained from the Gene Expression Omnibus repository (GSE121410). Unbiased 2-dimensional hierarchical clustering and heatmap visualization of differential expressed coagulation factor genes were performed using an Array Studio 10 (OmicSoft, Cary, NC).

### Human kidney single-cell RNAseq data analysis

The results here are in whole or part based upon data generated by the Kidney Precision Medicine Project (KPMP): DK114886, DK114861, DK114866, DK114870, DK114908, DK114915, DK114926, DK114907, DK114920, DK114923, DK114933, and DK114937. Data were downloaded from https://www.kpmp.org on 8/29/2022 (35). Downstream analysis was performed using the R package Seurat (v4.0.6) and figures were generated with functionalities “Dimplot,” “FeaturePlot,” and “VlnPlot” (36) To reduce cluster numbers, clusters were renamed based on the KPMP study (37) using Python 3.

### Isolation of BMMø and culture condition

Mouse bone marrow cells were flushed from the femur and tibia and cultured in L929 cell-conditioned medium to separate adherent differentiated cells for 6 days. The media was changed every 2 days to remove nonadherent, and immature cells. To achieve polarization of BMMø, BMMø were stimulated with Lipopolysaccharides (LPS) (Sigma-Aldrich) or IL-4 and IL-13 (BioLegend) for 24 h. For calcium treatment, 50 mM calcium chloride (CaCl_2_) was treated for 18 h and cell lysates were prepared.

### Statistics

Data represent the mean ± SEM prepared using GraphPad Prism 9.0 (GraphPad Software Inc., La Jolla, CA, United States). Statistical analyses were performed using the Mann–Whitney U test (one-tailed). The *p* values that were greater than 0.05 were considered significantly different. Statistically significant *p* values are denoted as *p < 0.05, **p < 0.01, and ***p < 0.001.

## Results

### Coagulation factors are upregulated in the kidney after kidney injury

**Figure 1:**
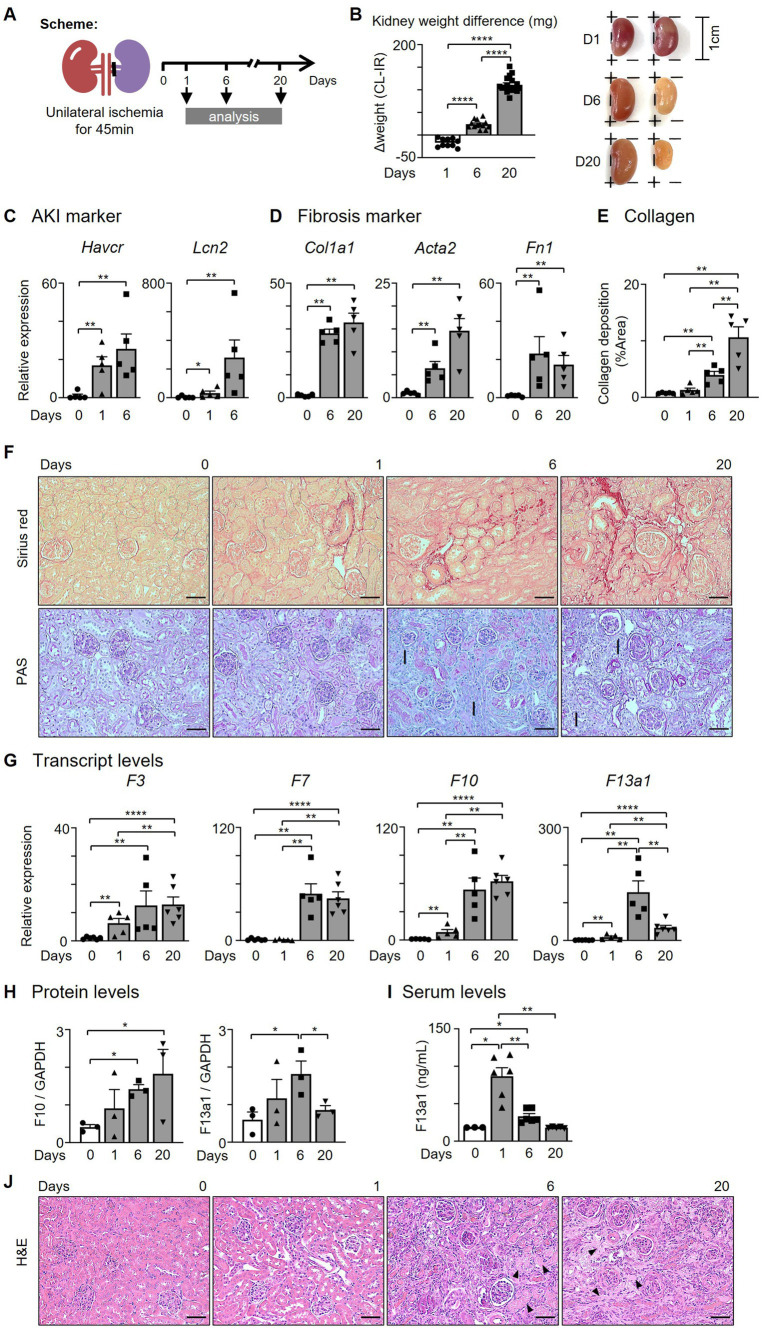
The levels of coagulation factors are increased in the kidney after I/R. **(A)** Experimental scheme. Unilateral kidney ischemia/reperfusion (I/R) surgery was performed for 45 min with C57BL/6 J female mice (6–8 weeks). **(B)** Kidney weight comparison between contralateral and I/R kidneys. **(C,D)** Expression of **(C)** AKI and **(D)** fibrosis genes after I/R surgery. **(E)** Quantification of collagen deposition by picrosirius red positive areas in the kidney after I/R surgery. **(F)** Representative pictures of picrosirius red and PAS staining of kidneys (Magnification 20x; Scale bar: 50 μm; I: interstitial fibrosis). **(G,H)** Expression of coagulation factors at 0, 1, 6, and 20 days after I/R surgery. Transcript and protein levels were assessed by **(G)** RT-qPCR (*n* = 5-6/group) and **(H)** western blotting (WB, *n* = 3/group). **(I)** Serum F13a1 levels were evaluated with ELISA (*n* = 3-6/group). **(J)** Representative pictures of H&E staining of the kidney (Magnification 20x; Scale bar: 50 μm; Arrows: fibrin matrix). Data are shown as mean ± SEM. *p < 0.05; **p < 0.01; ****p < 0.0001; Mann–Whitney U test.

We hypothesized that kidney Mø express coagulation factors, which contribute to the provisional matrix formation, after an acute kidney injury (AKI). To test our hypothesis, we employed unilateral kidney ischemia–reperfusion (I/R) surgery, a murine model of sterile AKI (Figure 1A). Kidneys were then analyzed on days 1 (AKI), 6 (transition phase), and 20 (fibrosis). I/R kidneys were enlarged on day 1 and shrank until day 20 of I/R compared to contralateral (CL) kidneys (Figure 1B). AKI genes (*Havcr* and *Lcn2*) were elevated on days 1 and 6, and fibrosis genes (*Col1a1*, *Col1a2*, *Col3a1,* and *Fn1*) on days 6 and 20 of kidney I/R (Figures 1C,D, Supplementary Figure S1A). To verify that I/R induces kidney fibrosis on day 20, picrosirius red and PAS staining were performed. Collagen deposition (picrosirius red) and structural changes, such as tubular atrophy, and intratubular cast formation, were indicative of kidney fibrosis in I/R kidneys at day 20 (Figures 1E,F).

Next, we determined whether the expression of coagulation factors is increased within the kidney following kidney I/R and found that intrarenal *F3*, *F7*, and *F10* transcripts were significantly increased until day 20 (Figure 1G). The expression of Intrarenal *F13a1* transcript peaked in the transition phase (day 6) (Figure 1G). The protein levels of F10 and F13a1 showed an expression pattern corresponding to transcript data (Figures 1G,H, Supplementary Figure S1B). To determine whether the upregulated protein level of F13a1 within the kidney is derived from the tissue or circulation, we probed for the serum level of F13a1 at different time points after AKI (Figure 1I). Serum F13a1 level peaked on day 1 and immediately decreased to the basal level by day 6, indicating that upregulated intrarenal F13a1 on days 6 and 20 of I/R is a tissue-specific response.

Since F13a1 is known as a fibrin stabilizing factor, which crosslinks fibrin filaments to make fibrin polymer and stabilize clots, we investigated whether the fibrin matrix is present as the F13a1 level increases with H&E staining (Figure 1J). As the arrows point, the fibrin matrix was prominent on day 20 of the I/R group. Taken together, our data suggested that the coagulation factors including F13a1 are expressed by the kidney tissue following kidney I/R.

### Intrarenal Mø subpopulations show distinct expression patterns and levels of coagulation factors

Our data indicated that the levels of coagulation factors (e.g., F10 and F13a1) are increased in the kidney after I/R. Next, we analyzed whether coagulation factors are expressed by kidney Mø found after I/R surgery.

**Figure 2:**
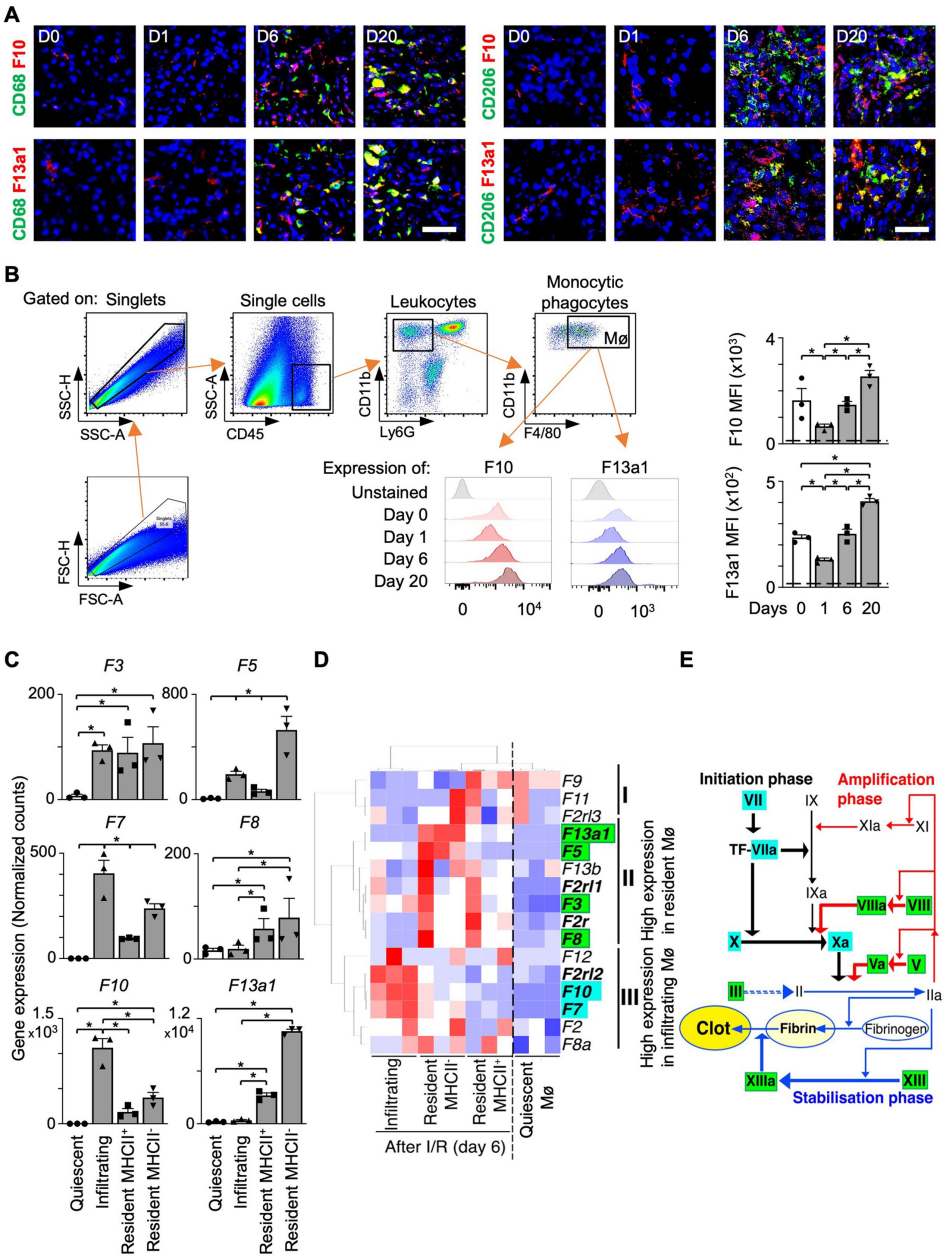
Intrarenal Mø subpopulations express distinct coagulation factors. **(A)** Kidney sections co-stained for an Mø marker (CD68 or CD206) and a coagulation factor (F10 or F13a1) (Magnification 20x; Scale bar: 25 μm). **(B)** Flow cytometry analysis of F10 and F13a1 expression by Ly6G-CD11b + F4/80+ cells. Dashed line: unstained cells (*n* = 3). **(C–E)** Differential gene expression data were obtained from the Gene Expression Omnibus repository (GSE121410). **(C)** Normalized RNAseq counts (FKPM) for coagulation factor transcripts in kidney Mø populations (*n* = 3). **(D)** Unbiased 2-dimensional hierarchical clustering and heatmap visualization of coagulation factor expression. **(E)** The relationship between coagulation factors and Mø subpopulations in the coagulation cascade (Roman: coagulation factors) Data are shown as mean ± SEM. *p < 0.05; Mann–Whitney U test.

We detected co-localization of Mø markers (CD68 or CD206) and F10 or F13a1 in the transition (day 6) and fibrosis phase (day 20) (Figure 2A, Supplementary Figure S2). In flow cytometry analysis, the mean fluorescence intensity (MFI) of F10 and F13a1 on Mø increased from day 1 to day 20 of I/R (Figure 2B).

**Figure 3:**
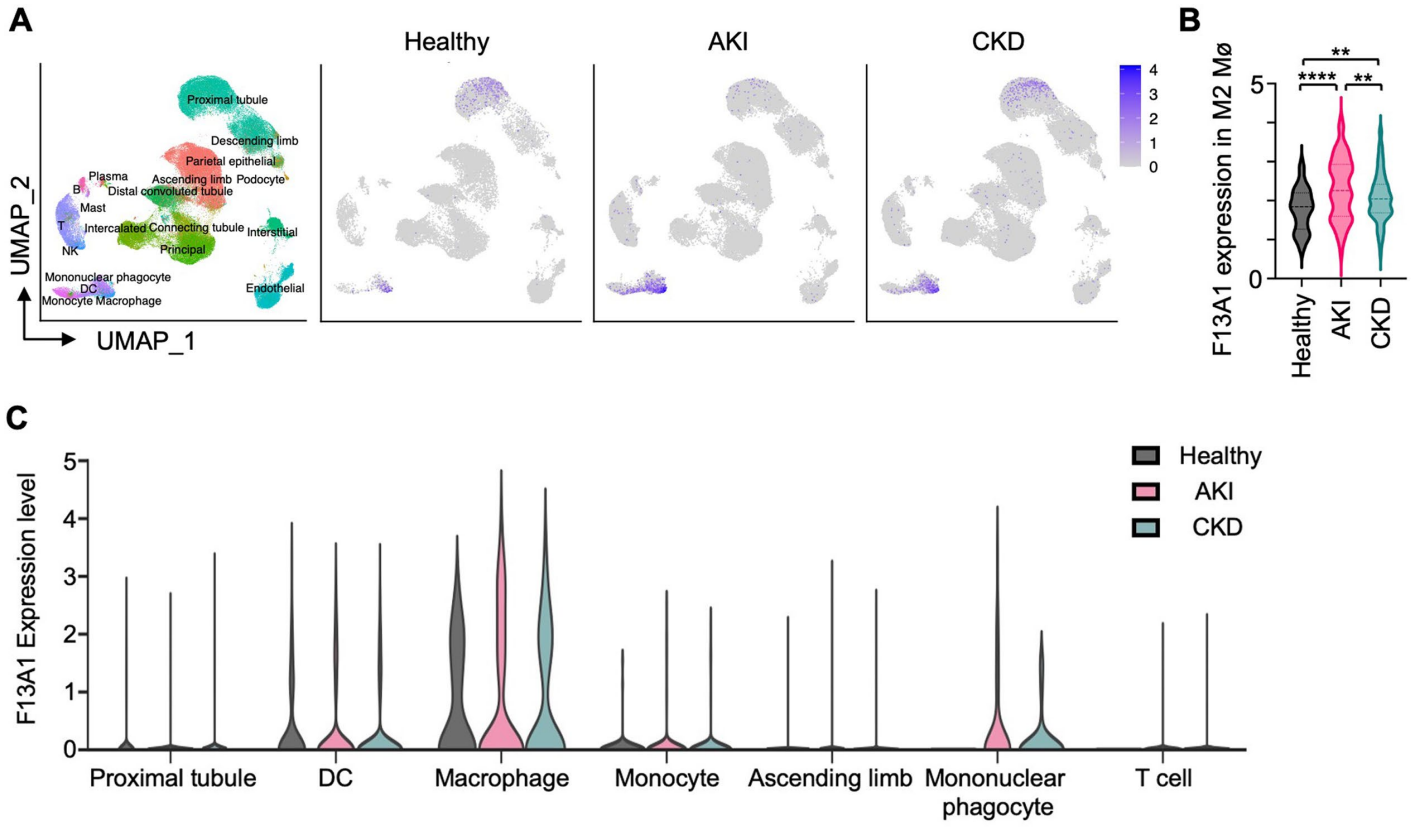
Mø are the most potent producers of F13A1 in human kidneys with AKI and CKD. Human kidney single-cell RNAseq data analysis. **(A)** UMAP represents 110,346 cells of 47 human samples (Healthy = 20, AKI = 12, CKD = 15) colored by cluster identity (*Left*). UMAP shows F13A1 gene expression in different kidney disease types (*Right*). **(B)** Violin plot showing F13A1 expression in F13A1 expressing M2 Mø. **(C)** Violin plot showing F13A1 expression level by highly expressing cell clusters. Data are shown as mean ± SEM. **p < 0.01. ***p < 0.001; Mann–Whitney U test.

Next, we questioned whether coagulation factors are expressed uniformly by all kidney Mø or only by specific kidney Mø subpopulations during kidney regeneration. Of note, I/R kidneys harbor a heterogeneous pool of Mø including infiltrating (Ly6C^high^), MHC II^+^, and MHC II^−^ resident subpopulations (24). To answer our question, we probed for the expression of coagulation factors by different kidney Mø subpopulations using RNA sequencing (RNAseq) data, which were generated from kidneys at day 6 of I/R (GSE121410) (Figures 2C,D). To our surprise, unbiased hierarchical clustering analysis revealed that infiltrating and resident subpopulations express coagulation factors, which were distinct: Infiltrating Mø (M1-like) expressed coagulation factors driving the initiation (e.g., *F7* and *F10*), whereas resident Mø (M2-like) produced factors responsible for the amplification of the coagulation cascade (e.g., *F3* and *F8*) (Figures 2D,E, Supplementary Figure S3). In this analysis, we additionally found that the coagulation factor most strongly upregulated by resident Mø is *F13a1*, which catalyzes the last step of coagulation by crosslinking fibrin molecules to fibrin clots (Figures 2C,E). Next, we sought to verify our findings from mouse RNAseq data in humans. To this end, we examined the expression of coagulation factors by Mø using human kidney single-cell RNAseq data (Supplementary Figure S3). Our results indicated that F13A1 is upregulated in M2 Mø from AKI and CKD patients (Figures 3A,B) and the main source of F13A1 expression in human kidney patients is Mø (Figure 3C). In humans, we could not detect other coagulation factors expressed in Mø. It is conceivable that the transient expression of coagulation factors, combined with the inherent differences between human pathologies and animal models, could account for this discrepancy. Nevertheless, our data suggests that Mø-derived F13a1 may contribute to fibrinogenesis and in-tissue clotting and affect the development of kidney fibrosis.

### Calcium (Ca^2+^) induces the expression of coagulation factors in Mø

**Figure 4:**
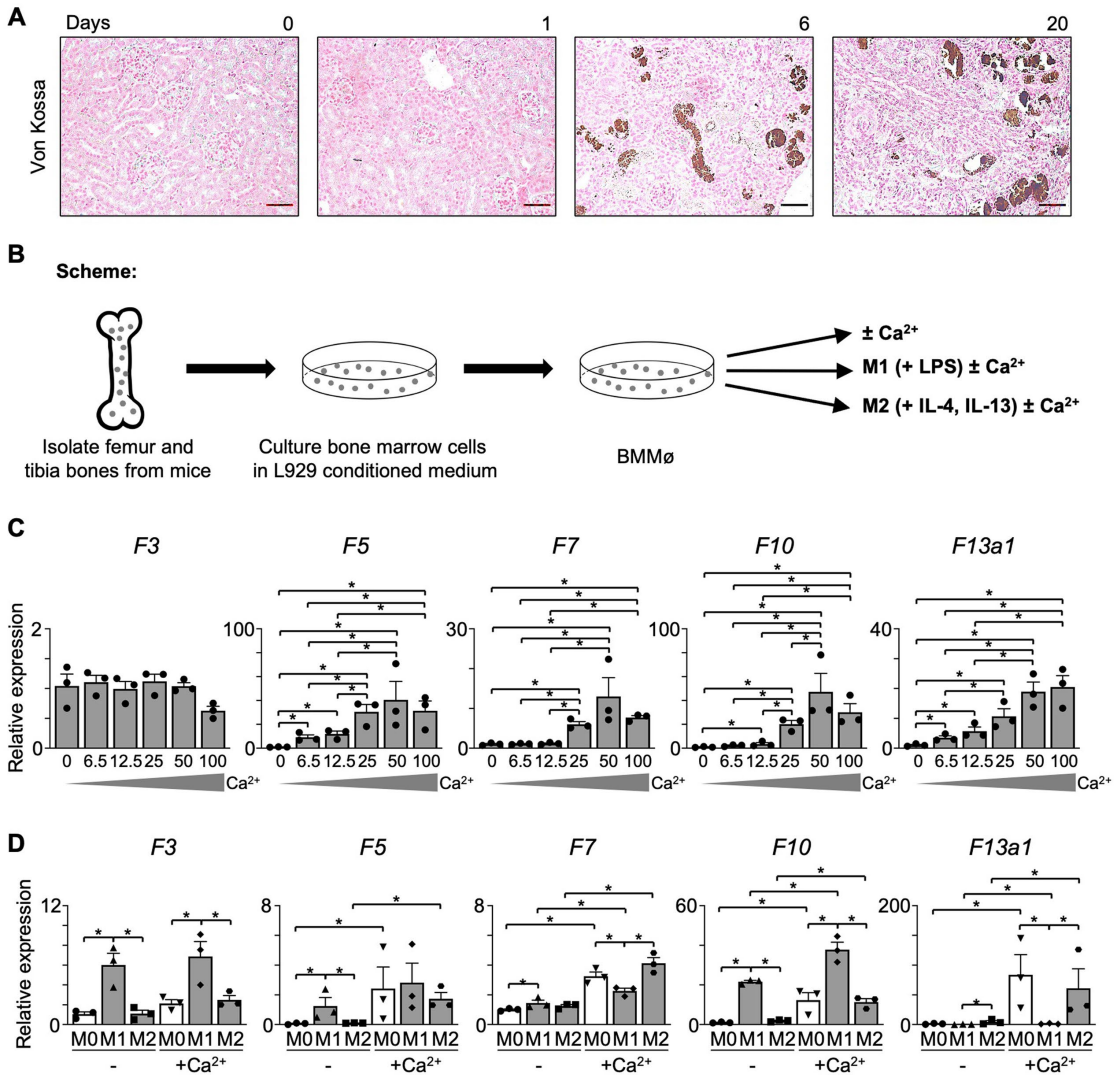
The expression of coagulation factors in Mø is dependent on Ca^2+^. **(A)** Von Kossa staining (Magnification 20x; Scale bar: 50 μm). **(B)** Experimental scheme. Bone marrow was obtained from C57BL/6 J mice and differentiated into Mø in an L929-conditioned medium. **(C)** Non-polarized (M0) BMMø were treated with Ca^2+^ in a concentration series. At 18 h of treatment, the level of coagulation factors was evaluated using RT-qPCR (*n* = 3). **(D)** BMMø were further differentiated into M1 Mø using LPS and into M2 Mø using IL-4 and IL-13 and treated with Ca^2+^ for 18 h. The expression of coagulation factors was evaluated with RT-qPCR (*n* = 3). Data are shown as mean ± SEM. *p < 0.05; Mann–Whitney U test.

Ca^2+^ plays an essential role in the coagulation cascade and is indispensable for the activation of several coagulation factors. In the conversion of prothrombin to thrombin, Ca^2+^ forms a complex with F10 and F5, forming the prothrombinase complex. We next probed for the presence of Ca^2+^ in I/R kidneys using von Kossa staining and found that Ca^2+^ is deposited adjacent to the proximal tubule and glomerulus in both transition (day 6) and fibrosis (day 20) phases, where Mø infiltrate (Figure 4A). This let us hypothesize that Ca^2+^ affects coagulation factor production in Mø.

To further explore the effects of Ca^2+^ on Mø, bone marrow-derived Mø (BMMø) were treated with CaCl_2_ for 18 h (Figure 4B). In a Ca^2+^ concentration series, we found that BMMø produce coagulation factors in presence of Ca^2+^ in a dose-dependent manner. The expression of *F5*, *F7*, *F10,* and *F13a1* in unpolarized (M0) BMMø increased dose-dependently until 50 mM, while F3 was not affected (Figure 4C). Also, M1 or M2 upregulated coagulation factors when treated with 50 mM Ca^2+^ (Figure 4D). *F3* and *F10* were significantly increased in M1 Mø, while *F7* and *F13a1* increased in M2 Mø upon Ca^2+^ treatment. Notably, the increase of *F13a1* expression after Ca^2+^ treatment was most prominent compared to other coagulation factors. Taken together, we can conclude that Ca^2+^ induces the upregulation of coagulation factors, especially that of *F13a1* in kidney Mø.

## Discussion

In this study, we tested the hypothesis that kidney Mø express coagulation factors during kidney injury. Here, we report that (1) both infiltrating (M1-like) and kidney-resident (M2-like) Mø produce non-redundant coagulation factors during AKI and CKD, which are key to fibrinogenesis; (2) *F13a1* is the most strongly upregulated coagulation factor in Mø in kidney I/R model as well as M2 Mø in AKI and CKD patients; (3) the upregulation of coagulation factors in Mø occurs in a Ca^2+^-dependent manner.

Our data provide many novel insights. Based on our data, we learn that (1) Mø are actively involved in fibrinogenesis and potentially in the subsequent fibrosis and should be considered effector cells of fibrosis. Mø are, at least, more important than any other renal cells in fibrinogenesis, i.e., provisional matrix formation (Figure 3C); (2) to our surprise, infiltrating (M1-like) Mø actively contribute to fibrinogenesis by expressing coagulation factors that drive the initiation of the cascade, implying that the current conceptualization of infiltrating Mø as anti-fibrotic cells must be reviewed (15–20); (3) tissue-resident Mø mediate the amplification and stabilization phase of the coagulation cascade, not being functionally redundant with infiltrating Mø; (4) Mø are the main source of F13A1 in the kidney during AKI and CKD. Interestingly, this finding is notwithstanding a study suggesting that F13A1 is not expressed in kidney-resident Mø (36). Our study clearly shows that F13A1 is expressed by renal Mø in both mice and humans. Recently, it has been shown that monocytes give rise to myeloid fibroblasts through M2 Mø polarization (38–41). It might be interesting to examine the expression of coagulation factors in this newly identified cell population.

Taken together, our data unveil Mø as a critical source of coagulation factors in fibrinogenesis and suggest the Mø-mediated intrarenal clotting process as a potential target for the treatment of fibrosis in the kidney and other organs. The increase of coagulation factors occurs dependently on Ca^2+^, which is abundantly present in the inflamed kidney (Figure 4A). Of note, this study provides the first evidence of the direct role of kidney Mø in fibrinogenesis and (provisional) matrix formation.

## Data availability statement

The datasets presented in this study can be found in online repositories. The names of the repository/repositories and accession number(s) can be found in the article/Supplementary material.

## Ethics statement

The studies involving human participants were reviewed and approved by Handong IRB. Written informed consent for participation was not required for this study in accordance with the national legislation and the institutional requirements.

## Author contributions

HO: data curation, investigation, and writing – original draft. OK: data curation, formal analysis, and software. MK: methodology. KP: resources. J-HB: conceptualization, data curation, funding acquisition, writing – reviewing and editing, and supervision. All authors contributed to the article and approved the submitted version.

## Funding

This work was supported from the National Research Foundation of Korea (NRF) through the Ministry of Education (2021R111A3059820) (to J-HB) and through the Ministry of Science and ICT (MIST) of R.O.K. (2020R1A2C2006903) (to KP).

## Conflict of interest

The authors declare that the research was conducted in the absence of any commercial or financial relationships that could be construed as a potential conflict of interest.

## Publisher’s note

All claims expressed in this article are solely those of the authors and do not necessarily represent those of their affiliated organizations, or those of the publisher, the editors and the reviewers. Any product that may be evaluated in this article, or claim that may be made by its manufacturer, is not guaranteed or endorsed by the publisher.

## Abbreviations

AKI: acute kidney injury
BMMø: bone marrow-derived macrophages
CKD: chronic kidney disease
CL: contralateral
F: coagulation factor
MFI: Mean Fluorescence Intensity
Mø: macrophage(s)
I/R: ischemia–reperfusion
