# Supplementary material for: Macrophages promote Fibrinogenesis during kidney injury
Source: Front Med (Lausanne). 2023 Jun 22;10:1206362. doi: 10.3389/fmed.2023.1206362 (PMC10325639; doi:10.3389/fmed.2023.1206362)
Supplement: Supplementary file 1 [file Data_Sheet_1.docx]

**Supplemental materials**

| Gene | FW/RV | sequence |
| --- | --- | --- |
| *Acta2* | FW | 5’-CCCAACTGGGACCACATGG-3’ |
|  | RV | 5’-TACATGCGGGGGACATTGAAG-3’ |
| *Col1a1* | FW | 5’-GCTCCTCTTAGGGGCCACT-3’ |
|  | RV | 5’-CCACGTCTCACCATTGGGG-3’ |
| *Col1a2* | FW | 5’-GTAACTTCGTGCCTAGCAACA-3’ |
|  | RV | 5’-CCTTTGTCAGAATACTGAGCAGC-3’ |
| *Col3a1* | FW | 5’-CCTGGCTCAAATGGCTCAC-3’ |
|  | RV | 5’-CAGGACTGCCGTTATTCCCG-3’ |
| *F3* | FW | 5'-AACCCACCAACTATACCTACACT-3' |
|  | RV | 5'-GTCTGTGAGGTCGCACTCG-3' |
| *F5* | FW | 5'-CGCAACTAAGGCAGTTCTATGT-3' |
|  | RV | 5'-GCTAGATCGTGGCTTTTCTTTCT-3' |
| *F7* | FW | 5'-AAAGGCGTGCCAACTCACTC-3' |
|  | RV | 5'-CCTACGTTCTGACATGGATTCG-3' |
| *F10* | FW | 5'-AGGACTCGGAGGGCAAACT-3' |
|  | RV | 5'-TCACGGACCTCTTCATAAGAACA-3' |
| *F13a1* | FW | 5'-GAGCAGTCCCGCCCAATAAC-3' |
|  | RV | 5'-CCCTCTGCGGACAATCAACTTA-3' |
| *Fn1* | FW | 5’-ATGTGGACCCCTCCTGATAGT-3’ |
|  | RV | 5’-GCCCAGTGATTTCAGCAAAGG-3’ |
| *Gapdh* | FW | 5'-AGGTCGGTGTGAACGGATTTG-3' |
|  | RV | 5'-TGTAGACCATGTAGTTGAGGTCA-3' |
| *Havcr* | FW | 5’-ACATATCGTGGAATCACAACGAC-3’ |
|  | RV | 5’-ACAAGCAGAAGATGGGCATTG-3’ |
| *Lcn2* | FW | 5’-GTTAAACCAGAGATTCCCACACG-3’ |
|  | RV | 5’-TCTCATGGGGACAAAATGTAGTG-3’ |

**Supplementary Table 1. Primers for Real-Time quantitative PCR (RT-qPCR)**

**
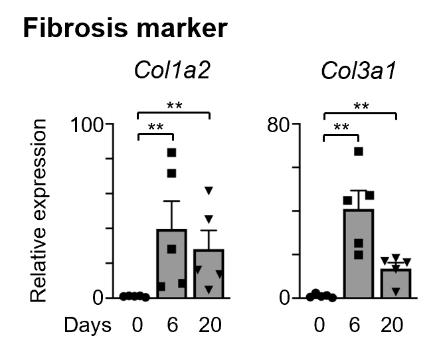
A**

 **B**

**Supplementary Figure 1. (A) Expression of fibrosis marker (*Col1a2*, *Col3a1*) at 0, 6, and 20 days after I/R surgery.** Transcript levels were assessed by RT-qPCR. *n* = 5-6/group. (B) **Original western blot pictures.** Whole un-cropped images of the original western blots shown in Fig 1H. Data are shown as mean ± SEM. **P*< 0.05. ***P* < 0.01. ****P* < 0.001; Mann-Whitney U test.

**Supplementary Figure 2. Immunostaining of kidney tissue with Mø marker and coagulation factor.**

Kidney sections co-stained for an Mø marker (CD68 or CD206) and a coagulation factor (F10 or F13a1) (Magnification 20x; Scale bar: 25 μm).

**Supplementary Figure 3. RNA-seq data for coagulation factor transcripts in kidney Mø populations.**

Differential gene expression data obtained from Gene Expression Omnibus repository (GSE121410). Normalized RNA-seq counts (FKPM) for coagulation factor transcripts in kidney Mø populations. *n* = 3. Data are shown as mean ± SEM. **P*< 0.05. Mann-Whitney U test.


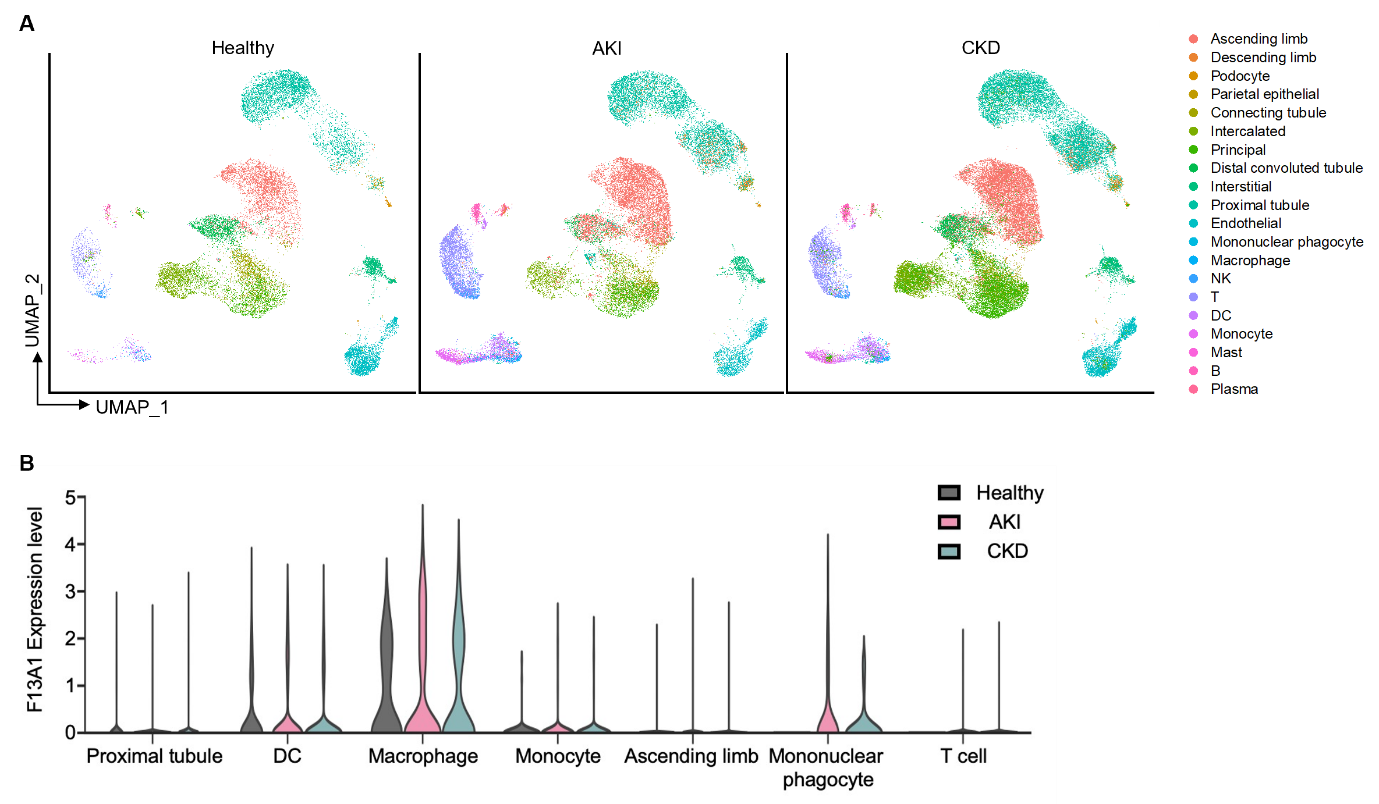


**Supplementary Figure 4. Human kidney single-cell RNA sequencing data analysis.**

UMAP represent total 110,346 cells split by kidney disease type (*n* = 21,463 Healthy; *n* = 35,627 AKI; *n* = 53,256 CKD).
